# Ecological Complexity in a Coffee Agroecosystem: Spatial Heterogeneity, Population Persistence and Biological Control

**DOI:** 10.1371/journal.pone.0045508

**Published:** 2012-09-20

**Authors:** Heidi Liere, Doug Jackson, John Vandermeer

**Affiliations:** 1 Department of Entomology, University of Wisconsin, Madison, Wisconsin, United States of America; 2 Department of Ecology and Evolutionary Biology, University of Michigan, Ann Arbor, Michigan, United States of America; University of Lancaster, United Kingdom

## Abstract

**Background:**

Spatial heterogeneity is essential for the persistence of many inherently unstable systems such as predator-prey and parasitoid-host interactions. Since biological interactions themselves can create heterogeneity in space, the heterogeneity necessary for the persistence of an unstable system could be the result of local interactions involving elements of the unstable system itself.

**Methodology/Principal Findings:**

Here we report on a predatory ladybird beetle whose natural history suggests that the beetle requires the patchy distribution of the mutualism between its prey, the green coffee scale, and the arboreal ant, *Azteca instabilis.* Based on known ecological interactions and the natural history of the system, we constructed a spatially-explicit model and showed that the clustered spatial pattern of ant nests facilitates the persistence of the beetle populations. Furthermore, we show that the dynamics of the beetle consuming the scale insects can cause the clustered distribution of the mutualistic ants in the first place.

**Conclusions/Significance:**

From a theoretical point of view, our model represents a novel situation in which a predator indirectly causes a spatial pattern of an organism other than its prey, and in doing so facilitates its own persistence. From a practical point of view, it is noteworthy that one of the elements in the system is a persistent pest of coffee, an important world commodity. This pest, we argue, is kept within limits of control through a complex web of ecological interactions that involves the emergent spatial pattern.

## Introduction

It is familiar knowledge that local interactions can sometimes create complex and surprising self-organized spatial patterns [1]–[3]. Spatial patterns from spots in animal coats to animal distribution patterns, have been shown to arise from simple local dispersion and interactions. For example, the distribution of species in simple predator-prey and parasitoid-host deterministic models in homogeneous environments can form spiral waves, clustered distributions, crystal lattices, and chaotic patterns [1], [2], [4], [5]. Furthermore, it has often been noted that spatial heterogeneity can allow coexistence of predator-prey and parasitoid-host associations that are otherwise prone to local extinctions [6]–[11]. In the present communication, stimulated by the natural history of a community of ladybeetles, ants, and scale insects in a coffee agroecosystem, we propose that the spatial heterogeneity–used here to describe two different habitat types with a specific spatial arrangement–needed for the persistence of a system might be indirectly caused by the dynamics of that very system. That is, the habitat conditions required for persistence of a population may be caused by the dynamics of that very population.

The heterogeneous habitat that allows beetle persistence is the clustered spatial distribution of a tropical arboreal ant species, *Azteca instabilis*, in a coffee agroecosystem [12]. This tree-nesting ant occupies about 3% of shade trees in a 45-ha plot, and its nests form a clustered distribution in the farm (Vandermeer et al. [12] defined two nests as belonging to the same cluster if they were less than 20 m apart). Given the lack of evidence for environmental factors to explain these patterns (i.e. they found no differences in tree species composition, tree size or canopy cover between areas with and without ant clusters), these authors hypothesized that the clustered spatial pattern could be explained by positive density-dependent spread of ant nests from tree to tree balanced by a negative density-dependent control from a natural enemy of the ant (parasitoid phorid flies (Diptera: Phoridae) from the genus *Pseudacteon*: *P. laciniosus*, *P. planidorsalis* and *P. pseudocercus* [13]). Given the demonstrated tendency of these phorid flies, to concentrate on large ant nest clusters, Vandermeer and colleagues [12] attributed the negative density-dependent force that is inhibiting ant nest clusters from unrestricted expansion to the direct effect of this parasitoid fly. Using a stochastic cellular automaton, these authors closely approximated the actual spatial pattern with this hypothesized dynamic. The resulting emergent spatial pattern creates significant habitat variability and thus a potentially influential force for any organism associated with *A. instabilis.*

The ladybeetle, *Azya orbigera,* is an example of an organism that might be strongly influenced by the spatial pattern of *A. instabilis.* This predatory ladybeetle is a voracious predator of the green scale, *Coccus viridis*, an important coffee pest. *Coccus viridis* maintains a mutualistic association with *A. instabilis* and is thus only highly abundant on coffee plants in close proximity (2–3 m) to ant nests [14]. For *A. orbigera,* this mutualistic association has two contradictory effects. On the one hand, adult ladybeetles are normally not permitted to eat in ant-tended areas due to the aggressive action of the ants [15]. On the other hand, given that the ladybeetle larvae are covered by waxy secretions that deter ant attacks, they are able to live and prey in ant-tended plants [16]. Furthermore, due to the ants’ generalized harassing behavior, natural enemies (predators and parasitoids) that normally attack the ladybeetle larvae are prevented from doing so (see Video S1). Thus, in areas with ants, larval beetles have abundant food and enemy-free space, while adults are almost completely prevented from eating. Contrastingly, in areas without ants patrolling the poorly-dispersing ladybeetle larvae are under considerable risk of starvation and pressure from their natural enemies, while adult ladybeetles are able to fly from plant to plant searching for sparsely distributed, non-tended scales. Indeed, field surveys in the area show that both beetle adults and larvae are significantly more abundant in areas with ants but that due to ant harassment adults are forced to disperse to areas without ants to feed (Liere et al. in preparation). Given this natural history, we suspected that the beetle population must live in a spatially heterogeneous environment with respect to the *A. instabilis* distribution in such a way as to provide adequate habitat for both larval and adult stages, the larvae requiring ant habitats to survive and the adults requiring ant-free habitats.

Thus, an ant/scale insect mutualism affected by phorid parasitoids that attack the ants could create a spatial pattern (as in Vandermeer et al. [12]) which provides a habitat background that permits the predatory ladybeetle to persist. However, the basic natural history of the system suggests the possibility of a distinct, more interesting, dynamic. The beetle itself, rather than the parasitic phorid fly, could determine the spatial pattern of the ant nests in the first place. As stated above, Vandermeer et al. [12] showed that the clustered spatial structure of ants can be attributed to the expansion of ant nest satellites controlled by a negative density-dependent force, which they hypothesized to be a result of a parasitoid phorid fly directly acting on the ant. However, the structure of their model included only a generalized density-dependent mortality term, which could be due to the phorid, as they suggest, but also could be due to other density-dependent forces. In particular, like many honeydew-collecting ants [17], *A. instabilis* may depend on the energy source provided by its mutualistic partners to survive; consequently, any organism that reduces the population of scale insects could increase the mortality of ant nests. Additionally, if this control force increases the likelihood of ant nest mortality in a density-dependent fashion (in other words, if this force has a tendency to be stronger in larger clusters of ant nests), it could also play a role in the cluster-forming spatial dynamics of ant nests. Consequently, given that *A. orbigera*, just like the phorid fly, responds in a density-dependent fashion to ant nest clusters (Liere et al. in preparation), and due to its voracity and ability to exploit ant-tended scale colonies [15], [16] we propose that this predatory beetle may be responsible for the formation of ant clusters.

Thus, our goal in this communication is to demonstrate the feasibility for a clustered spatial pattern of ant nests to emerge from the natural history of the ant/scale/beetle system alone. At the same time, we seek to determine whether this pattern could then prove to be essential for the survival of one of the species that plays a key role in generating it. While many studies have shown similar predator/prey pattern-generating mechanisms, and other studies have demonstrated that heterogeneous spatial patterns are necessary for the persistence of predator/prey systems, this is a novel situation where a predator indirectly causes a spatial pattern of an organism other than its prey, and in doing so, facilitates its own persistence.

Specifically, we constructed a spatially-explicit demographic model to address the following questions: 1) Starting from random distributions, can the beetle/scale insect dynamics generate a clustered spatial pattern of ant nests similar to that seen in nature and to the one emerging from the Vandermeer et al. [12] model? And 2) Is the consequent spatial pattern of the ant nests important for the persistence of the beetle?

## Methods

To answer these questions, we constructed a spatially-explicit model as a cellular automata (CA) to simulate the presence and absence of ant nests. We then coupled the CA model with three coupled map lattices to simulate the scale insects and the ladybeetle (adults and larvae) populations in the lattice.

To answer the first question, we started by randomly scattering ant nests, scale insects, and beetle larvae and adults over the lattice, with some of the parameters delimited by the natural history of the system. We then used a genetic algorithm (GA) to search for values for the remaining parameters (see below). Then, we qualitatively compared the spatial distribution of the ant nests resulting from our model with that found in the field. Additionally, we compared the frequency distribution of ant nest clump sizes with that obtained by the Vandermeer et al. [12] model.

To answer the second question, we ran the model with the GA-fitted parameters and simulated four different scenarios: (1) we fixed the ant nest mortality parameters so that the ants went extinct; (2) we fixed the ant expansion parameters so that ant nests covered the entire lattice; (3) we modified the beetle migration parameters in order to eliminate the ability of the beetles to ‘perceive’ and respond to the emergent ant nest clusters; and (4) we ran the model so that the beetle contributed to the emergence of ant nest clusters, and this time allowing the beetles to perceive and respond to the clusters. We then followed the scale and beetle populations over time to determine their population persistence in the different scenarios.

### Model Description

The research that led to our understanding of the natural history of the system took place in a traditional shaded coffee plantation in southern Chiapas, Mexico (for review see [18]). Our model is intended to capture the essence of the biological interactions that create spatial structure and allow a beetle population to persist in this coffee agroecosystem without including any unnecessary details (the model pseudocode can be found in Methods S1). Therefore, our emphasis was on simplicity and not on all of the known aspects of the natural history of the system.

As with the model in Vandermeer et al. [12], our model uses a 120 cell X 90 cell grid with periodic boundaries to represent a 800 m×600 m study plot. Each of the four populations– ants, scale insects, beetle larvae, and beetle adults – is represented using a separate 120 cell X 90 cell grid. Each cell in these grids represents a shade tree (where the ants nest) and its neighboring coffee bushes (where the scale insects live and the beetles forage).

Since we were interested in the effects of ant presence and absence, the ant nest grid in our model contains only zeros and ones representing the presence or absence of ant nests, respectively. By contrast, each cell in the scale insect grid has a number representing the population size at each time-step and likewise for the beetle larvae and beetle adults.

### Process Overview and Scheduling

The model operates in discrete time and uses synchronous updating. Each time step corresponds to a six-month period, which matches the frequency of field surveys [12]. In accordance with the natural history of the system) this formulation assumes that larvae either eclose or die in one time step, and all adult beetles live only a single time step (under field conditions, the time from larval emergence to pupation is less than two months and adults survive an average of 4 months).

#### Model parameters

We employed a genetic algorithm implemented using the Java package JGAP (Java Genetic Algorithms Package) to search for parameter values that could generate model behavior consistent with the known characteristics of the natural system [19] (for details, see Methods S2). Our goal was to determine if it was possible, given the natural history of the system, to find parameter values that would result in the hypothesized dynamics: the emergence of ant nest clusters due to the indirect effect of beetle predation and the requirement of this emergent spatial pattern for the beetle survival. Thus, the performance (fitness) of parameter value combinations was calculated based on the following criteria: number of ant nests, complete extinction of beetles when ants are fixed to occupy 100% of the lattice points, complete extinction of beetles when ants are fixed to occupy 0% of the lattice points, numbers of beetle adults and larvae in locations with ant nests, and numbers of beetle adults and larvae in locations without ant nests (see Table S1). The target values for the numbers of ant nests, adults, and larvae were obtained from field data. As detailed in the following sections, certain parameter values were constrained based on the known biology of the system, e.g., the survivorship of beetle larvae in locations without ants was constrained to be less than the survivorship with ants.

In order to find acceptable parameter values more rapidly, the GA was run on 12 computers simultaneously. Each instantiation of the GA had a population of 100 possible solutions, with the parameter values in the first generation chosen at random (the maximum fitness per generation for each of the 12 instantiations is shown in Fig. S2). There was significant variability in the speed of the computers used, so there was substantial variation in the number of generations the machines completed before the search was terminated. The model, in the end, is quite parameter heavy, precluding a full sensitivity analysis; however, all of the GA instantiations converged rapidly to similar marginally acceptable or acceptable outcomes, suggesting that the range of acceptable parameter values is relatively large, i.e., the model output is relatively robust to changes in parameter values.

#### Interactions

Interspecific interactions in this model include: 1) the predator-prey relationship between beetle larvae and scale insects; 2) the predator-prey relationship between beetle adults and scale insects; 3) the mutualism between ants and scale insects, which is incorporated, on the one hand, in the increased ant nest mortality with decreased scale insect density and, on the other, in the increased intrinsic growth rate of scale insects and the reduced predation pressure suffered by scale insects in the presence of ants; 4) the inadvertent protection of beetle larvae by ants, which is incorporated in the ant nest-dependent survivorship rates of the larval beetles; and 5) the inhibitory effect of ants on beetle adults, which is incorporated in the ant nest-dependent consumption and survivorship rates of the adult beetles. Conspecific interactions are implicit in 1) the logistic growth rate of the scale insects, 2) the density-dependent expansion of ant nests, and 3) the density-dependent migration rates of scales and beetle adults.

The model starts with randomly scattering ant nests (ones and zeros), scales and beetles over the lattice (for details see pseudocode in Methods S1) and then iterates over the following five steps:

#### 1) Beetle population growth

For each iteration of the model, we first allow the beetles to reproduce and eat scales according to the cell type (with or without ants) with a Holling type II functional response:
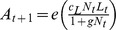


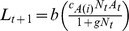
where each time step or generation time coincides with the six month sampling of ant nests we have from the field; A_t_ and A_t+1_ represent the beetle adult population at time t and t+1 respectively; L_t_ and L_t+1_ represent the beetle larvae population at time t and t+1 respectively; N_t_ is the population of scale insects at time t; e represents the eclosion rate (from larvae to adults); c_L_ is the consumption rate of larvae; g is the Type II functional response term for both larvae and adults, i.e. 1/g is the maximum predation rate per unit of time; b is the beetle birth rate; c_A(i)_ is the consumption rate of adults and according to the natural history of the system adult consumption rate of scales is smaller in patches with ants (c_A (A)_) than in patches without ants (c_A (noA)_) [15]. In contrast, the larval consumption rate of scales (c_L_) is not affected by the presence of ants [16]. Thus in the first step of our model the population of beetle adults in time t+1 depends on the larvae to adult eclosion rate (e) and on the consumption rate of the larvae at time t. The larval population at time t+1 depends on the birth rate (b) and on the consumption rate of adult beetles at time t.

#### 2) Ant nest mortality, ant short- and long-distance migration

These are all modeled probabilistically following the Vandermeer et al. [12] model with the exception of the mortality effect. Here we formulated the probability of mortality of an ant nest as a decreasing linear function of the local population of the scale insect. Thus, if a population of scale insects in a given cell is low, the probability of ant nest mortality is higher than for a cell where the population of scale insects is high. Short-distance migration reflects the satellite ant nest expansion that occurs in the field, and is modeled probabilistically as a linear function of the number of occupied cells in the Moore neighborhood (the surrounding 8 cells). Long-distance migration, which was added to reflect ant queen nuptial flight and new nest formation in the field, was modeled stochastically as propagule rain.

#### 3) Beetle and scale insect local and long-distance migration

We included stochastic migration of adult beetles both locally to the Moore neighborhood and globally as propagule rain. For local migration, the larger the local population (in the Moore neighborhood) of adult beetles, the higher the probability a given cell will receive migrants (a proportion of the adult beetle population from neighboring cells). For global migration, the likelihood that each cell received migrants was also stochastically determined and, according to the demonstrated ability of beetles to perceive ant pheromones [20], a cell with ants had a higher probability of receiving migrants than a cell without ants. ‘Chosen’ cells then received a proportion of the total adult beetle population in the whole lattice. We applied the same rules to scale insect local and global migration; however, since unlike adult beetles, scale insects in the field exhibit a ‘true’ propagule rain in the sense that they are dispersed by wind currents and land randomly in the landscape [21], their dispersal did not have a preference towards cells with ant nests in our model.

#### 4) Scale local dynamics

Scale population size is determined by equations inspired by a Rosenzweig-MacArthur model (with K = 1):
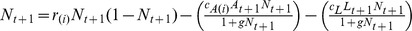
where the intrinsic rate of increase of scale insects, r_(i)_, was slightly lower in cells without ants as suggested by field data [22]. Thus, scale insects in our model have a logistic growth and experience death rates that depend on predation terms already described for the beetle adults and larvae.

#### 5) Beetle differential survival

Adult mortality:
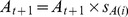



Larval mortality:
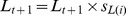



Given the natural history of the system, the proportion of surviving adults in cells with ants (s_A(A)_) is smaller than the proportion of surviving adults in cells without ants (s_A(noA)_); while the opposite is true for the proportion of surviving larvae (s_L(i)_).

After this, the number of ant nests and the population size of scales and beetles was calculated and the spatial distribution was plotted.

### Comparing the Models and Field Data

To compare the model output with the field data, we examined two indicators of spatial pattern: first, the approximation to a power law distribution of cluster sizes and second, clustering of ant nests as measured by Ripley’s K [23]. The power law fitting of field data and of our simulation results was performed principally for comparison purposes; our objective was not to suggest that the power law was the best possible fit for the cluster size distribution but rather to be able to contrast our simulations/field data comparison with that showed by the Vandermeer et al. [12] paper.

To calculate Ripley’s K, the number of other nests in the neighborhood of each nest is compared with the number expected for a random (Poisson) distribution. The neighborhood is defined by a sampling circle with a specific radius. To determine the degree of spatial clustering at different spatial scales, Ripley’s K is calculated for a range of sampling circles. Deviations from the random expectation indicate that the spatial pattern is either more clustered or more uniform than random, depending on the direction of the deviation [23].

We calculated Ripley’s K for the Vandermeer et al. [12] model, for the beetle model with the GA-fitted parameters, and for two sets of field data (as in their model, the rainy season 2004 and dry season 2004). To show the variability due to model stochasticity, we estimated 95% confidence limits calculated from 200 realizations of the two models. Additionally, we included 95% confidence limits for 200 random distributions generated using 282 nests (this is the number of nests present in the 2004 rainy season, which gives more conservative, i.e., larger, confidence intervals than using the 384 nests present in the 2004 dry season).

### The Four Scenarios

In the first scenario, we ran the model with the GA-fitted parameters but we modified the ant mortality parameter so that the ants became quickly extinct. Likewise, for the second scenario, we modified the expansion probability of the ants so that they quickly occupied the whole lattice. In the third scenario, we modified the beetle migration parameters so as to only allow them to migrate as propagule rain (probability of local migration = 0; probability of global migration = 1). The elimination of local migration in the simulation prevented the beetles from building up their populations in response to the clustering effect of the ants. Consequently, they responded only to the global populations and migrated accordingly. Lastly, we ran the model as described in the previous sections with the GA-fitted parameters so that the beetles contributed to the emergence of ant nest clusters and, this time, the beetles ‘perceived’ and responded to those clusters.

## Results

**Figure 1:**
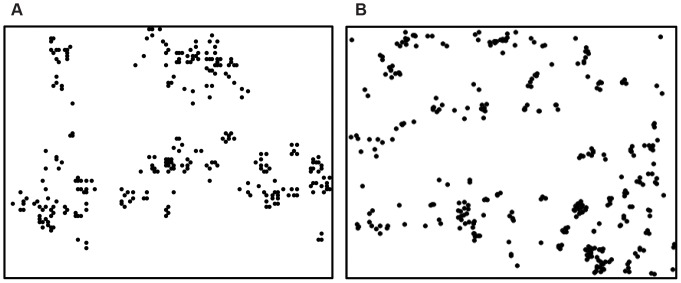
Field vs. simulation snapshot of the distribution of ant nests. A) From the 120×90 lattice from the theoretical model. B) From a 45 ha plot field survey conducted in the summer of 2006, qualitatively similar to the model results (for methods see Vandermeer et al. 2008). We use this size lattice as in their model, since the study system in nature is approximately that size and contains about 11,000 potential ant-nesting sites (shade trees).

**Figure 2:**
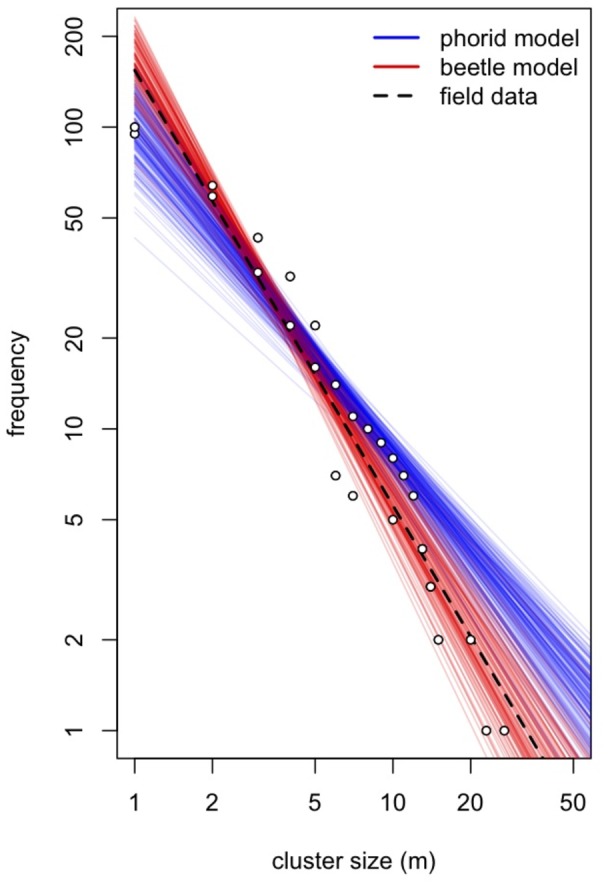
Log of cumulative frequency vs. log cluster sizes of ant nests (for easier interpretation, the axes have arithmetic scales). The open circles represent field data from 2004 (rainy and dry seasons) and the dashed line is the combined power law fit to both field surveys. Cluster size is based on a minimum distance of 20 m between nests, when these are judged to belong to the same cluster (see Vandermeer et al. 2008). The blue lines represent the fitted power law lines to each of 200 runs for the Vandermeer et al. 2008 model where a parasitic phorid fly is the cause of density dependent ant nest mortality. The red lines represent the power law fits to each of 200 model runs, where the coccinellid beetle is the indirect cause of ant nest mortality.

Starting from a random distribution of ants, scale insects, and beetles, and using the parameters found with the GA (see Methods S2, Table S2), the clustering of nests that emerges from our model is similar to that observed in the field (Fig. 1). Thus, casting the beetle population as an indirect density-dependent negative force indeed produces qualitative patterns that are similar to those observed in the field and reported by Vandermeer et al. [12]. Like their model, ours also produces a distribution similar to the field data, with a close fit to a power function (Fig. 2).

**Figure 3:**
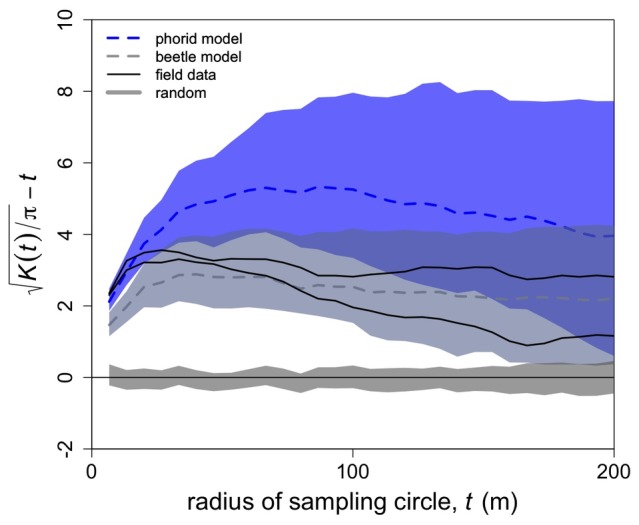
Ripley’s K index versus size of sampling circle around each tree. The blue color represents the results from the phorid model (Vandermeer et al. 2008) (the dashed line is the median and the shaded area shows the 95% confidence limits calculated from 200 realizations of the model); the slate blue color represents the results from the beetle model (200 realizations with the GA-fitted parameters); the dark gray shaded region represents the 95% confidence limits for 200 random distributions generated using the 2004 rainy season field data. The solid black lines are the results from rainy season and dry season field surveys performed in 2004. Deviations above the zero line indicate the data are more clustered than expected by random.

The profile of Ripley’s K-function, transformed such that the expectation for all spatial scales is zero for a random spatial pattern and greater than zero for a clustered pattern [24] (Fig. 3) suggests that the field data might be better explained by the Vandermeer et al. [12] model than by the beetle model at short spatial scales, and vice versa for large spatial scales. Thus, when the sampling circle is between 0 and approximately 25 m, the data closely approximate the pattern from the phorid model while between 25 and 75 m the beetle model gives a better fit. Above 75 m, the overlap of the two models does not allow them to be distinguished statistically.

**Figure 4:**
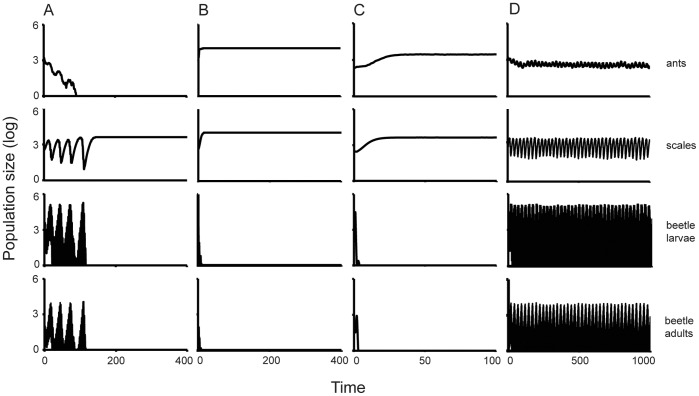
Simulation time series of the four populations (ants, scales, larvae beetles and adult beetles). Population sizes on the y-axis are on a log+1 scale. A) Simulation in which the ant population goes extinct (ant mortality parameters: d_o_ = 0.85; d_1_ = 0). B) Simulation in which the ant population occupies the whole lattice (ant mortality parameters: d_o_ = 0.2; d_1_ = 0.3). C) Simulation in which the ant population is similar to the one observed in the field, the beetles however, are not able to ‘perceive’ the ant clusters (local migration parameter: 0; global migration parameter = 1). D) Simulation in which the ant spatial pattern emerges from the model interactions and is similar to the one found in the field.

In the end, our model corresponds quite well with our expectations based on general field observations. The underlying dynamics generate, for some parameter values, the expected qualitative result that the beetles go extinct when the ants are either absent or cover the whole landscape, but are able to persist over the long term when the ant nests form a clustered distribution. Thus, as shown in Fig. 4, we were able to recreate the hypothesized dynamics based on the natural history of the system (with the parameter set displayed in Table S1): the beetles were only able to persist when the ant nests formed a spatial pattern which was, in turn, indirectly caused by the beetles themselves. The GA algorithm was able to efficiently find a set of parameter values that produced all the scenarios (Methods S2, Fig. S2). When the ant mortality parameters were set such that the ants went extinct, the beetle population crashed and the scale insects quickly reached their carrying capacity. Similar results were obtained in the second scenario, where the ant expansion parameters were set so that ant nests quickly occupied 100% of the lattice points. Finally, and most importantly, when the beetles were unable to perceive the ant nest clusters and responded only to global densities, the beetles quickly went extinct followed by the loss of the clustered pattern of the ant nests and an explosive increase of the scale insect populations.

## Discussion

Our model provides a conceptual representation of a subset of the key interactions in the system, and a possible explanation for both the emergence of spatial clustering and the persistence of the interacting species in the field. It is feasible that some abiotic or microclimatic condition that we have not yet measured could be driving the ant nest spatial pattern. We have, however, not been able to find any significant relationship between tree species, tree size or canopy cover and the formation of ant nest clusters (see Supporting Information for [12]) and given the uniform manner with which the whole farm is managed (in terms of soil fertility and shade cover), we strongly believe that the clusters are indeed caused by biotic interactions. Just as Vandermeer et al. [12] showed that with very simple local interactions a clustered pattern of ant nests similar to the one in the field could be generated, we showed that with an implicit density-dependent controlling force (i.e., the beetles eating the scale insects) a qualitatively similar pattern of ant nests can emerge for a feasible range of parameter values. Specifically, our model simulations show that starting with all the populations randomly distributed, ant satellite expansion coupled with predation pressure by the beetle on scale insects can form a clustered distribution of ant nests. At the same time, the existence of areas with and without ants created by this emergent spatial pattern was key to the persistence of the predatory beetle itself. Thus, we propose that through a complex network of interactions the beetles might be helping to create ant clusters that, in turn, provide the habitat heterogeneity necessary for their own survival.

Since our model was intended to produce qualitative results, the details of the parameters are not that important, and therefore a full sensitivity analysis was not performed. However, the speed at which the multiple GAs found parameter values that converged to an acceptable performance of our model, suggests that the model output is relatively robust to changes in parameter values (for details see Methods S2, Fig. S2). Although here, as in all parameter fitting exercises, it is impossible to know that our results are not derived from a local maximum, it is generally considered that a way of minimizing that likelihood is through the use of a genetic algorithm [25]. Given that we ran 12 different realizations of the genetic algorithm and they all converged on a similar fitness (see Fig. S2), it is unlikely that other, more fit, maxima exist.

**Figure 5:**
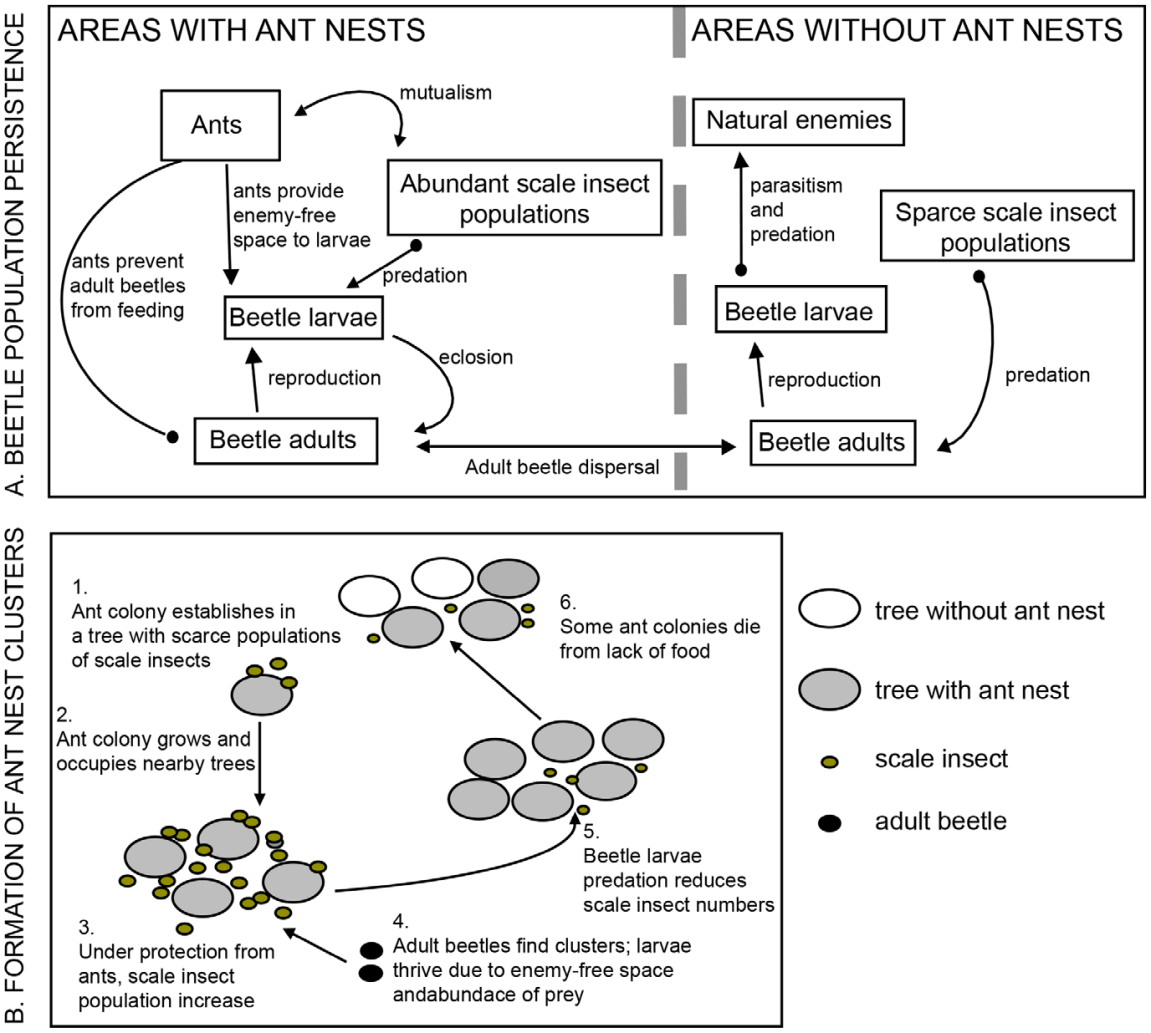
Diagrammatic representation of the proposed mechanisms allowing beetle population persistence (A.) and the formation of clusters of ant nests (B.). Arrowheads indicate positive effects, closed circles indicate negative effects.

In our model, the beetle acting as an indirect cause of ant nest mortality is the controlling force that counteracts the expansion of ant nests by satellite nest formation and contributes to the formation of the clustered spatial distribution of ant nests (Fig. 5B): The scale populations in newly colonized areas by ant nests can increase to relatively high values compared with areas with no ants, probably because of the combined action of *A. orbigera* (explicit in our model in the predation term) and other natural enemies. In areas with ant nests, where the protection by ants excludes most other natural enemies except *A. orbigera* (mostly the larvae), scale populations thrive and grow in size. This is followed by an increase of the beetle population, which eventually imposes a sufficient predation pressure to cause scale populations to decrease. These dynamics leave ants without their main carbohydrate source, and in doing so, increase their probability of mortality. Because of the build up of beetle populations in areas with ants, these areas act as sources for beetle adults that then disperse to the rest of the farm and contribute (most likely in conjunction with other predators, pathogens and parasitoids) to the maintenance of low scale insect populations. Due to the tendency of beetles to concentrate in areas with large clusters of ant nests (Liere et al. in preparation) and the diffusive nature of beetle migration, there is a stronger effect of beetle predation in larger clusters of ant nests, resulting in a density-dependent effect. The ability to respond in a density-dependent fashion to ant nests renders this beetle not only potentially important for the formation of ant nest clusters [12] but also sets up one of the most important traits of successful natural pest controllers, namely being able to concentrate in areas of high pest density [11].

A central assumption of the model is that beetles are able to decrease scale insect populations, indirectly increasing ant nest mortality. Some indirect evidence does suggest that this is possible in the field. First, we know that while *A. orbigera* is not the only natural enemy of *C. viridis* in the area, it is a voracious predator during both its larval and its adult stages. An individual beetle is able to consume an average of 25 scales per day, or as many as 600 scales as a larva and 1200 as an adult (Liere unpublished data). Given that in the field, on average, there are 3.8 larvae per coffee bush in areas with ants and 5.8 adults in the peripheries of these areas, beetles could indeed be a strong population control for the scale insects (Liere et al. in preparation). Second, while *A. instabilis* could conceivably survive on alternative carbohydrates and extrafloral nectaries, thus not depending solely on honeydew from *C. viridis*, only a few of the shade trees in the farm have extrafloral nectaries and populations of other species of scale insects are not as abundant as *C. viridis* (personal observations). Furthermore, a short-term study in the area suggests that *A. instabilis* colonies shift their foraging area in response to high levels of *C. viridis* mortality [26]. Thus, although we do not propose that the beetle is the sole driving control force in the field (other candidates being the phorid flies as Vandermeer et al. [12] proposed, and an entomopathogenic fungus that attacks the scale insect as proposed in Jackson et al. [26]), we suggest that given its voracity and natural history, the beetle could easily decrease scale populations so as to increase the probability of *A. instabilis* mortality. Furthermore, we suggest that given the tendency of beetles to aggregate in larger clusters of ant nests (Liere et al. in preparation), the beetle could also be a density-dependent force driving the formation of the clusters of ant nests, just as Vandermeer et al. [12] show for the phorid flies.

The result that *A. orbigera* requires areas with and without *A. instabilis* to survive depends on the assumption that *A. orbigera* is a specialist consumer of *C. viridis.* First, while it is true that *A. orbigera* adults, like the majority of ladybeetle species, are able to survive feeding on alternative prey [27], most other species of scale insects are generally scarce throughout the farm and even if tended by ants (*A. instabilis* or other ant species) they are never as abundant as *C. viridis* (personal observation). Second, while ladybeetle adults could survive for a while feeding on extrafloral nectaries, nectar or pollen, in the absence of their preferred or ‘essential’ prey, coccinellid beetles are not able to properly reproduce and larval mortality is very high [27], [28]. Thus, we believe that even if not strictly a specialist on *C. viridis*, this resource is important for the beetle’s proper growth, development and reproduction. This suggests that the existence of habitats with and without ants, as well as adult dispersal between habitat types, is essential for the beetle’s population persistence (Fig. 5A).

In reality, a combination of different forces is likely acting directly and indirectly on ant nest mortality in the field. For example, when comparing the frequency distribution of ant nest cluster sizes resulting from our model with the one generated by the Vandermeer et al. model, we see that the field data fall somewhere between the pattern predicted by the two models. This result suggests that both density-dependent forces, i.e., the beetle predation of scales and phorid fly parasitism, may be involved in the creation of the pattern. Accordingly, evidence gathered from the field strongly suggests that the phorid and the beetle are behaviorally interactive with one another [15]. In fact, by a reduction of ant activity, the presence of the phorid fly indirectly facilitates the consumption of scales by the adult ladybeetle and is also thought to increase the oviposition chances for female beetles in ant-tended areas (Liere et al. in preparation and [20]), supporting a synergistic role of these two forces in pattern formation and species persistence.

Furthermore, the Ripley’s K-function suggests that the field data correspond more to the phorid model than to the beetle model at short spatial scales and more to the beetle model at larger scales. The different ways in which the density dependence appears to operate both in the field and in the two models probably explains why the models seem to correspond to the field data at different scales. In the phorid model, density-dependent mortality is local (operating strictly in the Moore neighborhood of a nest). In contrast, concentrations of beetles that build up near ant nests can diffuse into the surrounding area through local dispersal, thereby reducing scale populations and affecting ant nest mortality over a larger area. Due to the overlapping influences of neighboring nests, this effect is stronger in larger ant clusters. Given that in nature the two forces are likely acting in conjunction and their effects might be non-additive, the comparison between the two models is in no way conclusive. Interestingly, however, the result coincides with what happens in nature, where the phorid flies directly affect ants locally by forcing them to reduce their tending and foraging activities [29], [30] while the aggregation (and hence predation) of beetles decreases more slowly with increasing distance from an ant nest, thus indirectly affecting the landscape of the ants at a larger scale.

Our various scenarios represent, first, two extreme cases, one with no ant nests and another with ant nests everywhere. Second, we simulated two scenarios where the number and spatial pattern of ant nests were similar to what is found in the field. The beetles, however, were only able to survive when they were able to perceive and respond to the clustered pattern of ant nests that was generated due to the indirect effect of the beetles themselves. Field evidence suggests that this spatial pattern is indeed important for the beetles’ persistence. The existence of large clusters of ant nests appears to be crucial for beetle oviposition, while small clusters of ant nests appear to improve larval survival (Liere et al. in preparation).

Not surprisingly, when the beetles went extinct in our model, the scale insects almost doubled their densities. Although we do not have direct evidence of the potential consequences of an extinction of beetle populations in the field, we believe that after being released from the controlling effect of such an abundant and voracious predator, the likelihood of scale insect outbreaks would increase, as our model results suggest.

There have been several theoretical studies reporting that predators (or parasitoids) can cause the formation of high and low prey-density patches in a homogeneous environment. In turn, prey spatial heterogeneity has been shown to be essential for the regional persistence of the predator/parasitoid population [31]–[35]. What is new about our system is not that a predator-prey system is able to persist by an emergent spatial pattern, but that the predator can indirectly generate a spatial pattern of an organism other than its prey, and that this pattern, in turn, is required by the predator for reasons other than variability in prey densities. A predator persists because the mutualistic partner of its prey is spatially structured and this spatial structure is only possible if the predator persists. Given the ubiquity of indirect interactions in biodiverse communities, we speculate that similar empirical examples may be common but have been widely overlooked.

From a practical point of view, the persistence of important natural enemies in agricultural systems is key for the natural control of potential pests. Furthermore, studying the details of arthropod food-webs in agricultural systems and consequent spatial distributions is particularly important since in order to keep herbivores below damaging levels, predators have to have the ability to aggregate in high density patches [36], [37], as is the case with *A. orbigera*. Furthermore, our results challenge the apparently straightforward implication that ants have a damaging effect to the plantation by protecting potentially harmful pests from their natural enemies [38], [39]. In fact, our results suggest that ants are necessary for the persistence of the important predatory ladybeetle and thus for the population control of green scales in the farm. Given that the scale insect is a persistent pest of coffee in many coffee-producing areas in the world, its maintenance below damaging levels in this particular farm may be an example of an important ecosystem service provided by complex local and spatial dynamics. From a theoretical point of view, the study of the effects of autonomously-created spatial patterns could shed light on the mechanisms that make biodiversity essential for the provisioning of ecosystems services and help explain how biodiversity enhances persistence and stability in biological communities.

## Supporting Information

Figure S1 **Flow chart of model execution.**
(TIF)

Figure S2 **Fitness versus generation for 12 instantiations of the Genetic Algorithm.**
(TIFF)

Table S1 **Genetic Algorithm fitness function coefficients.**
(DOCX)

Table S2 **Parameter values used for the model.**
(DOCX)

Methods S1 Model pseudo-code.
(DOCX)

Methods S2 Genetic algorithm (GA) description and parameters.
(DOCX)

Video S1 **Ant scaring away the beetle parasitoid.** The movie shows a parasitoid wasp (*Homalotylus shuvakhinae*, Encyrtidae, Hymenoptera) on the top of a leaf. Its host, the beetle larvae is at the apex of the leaf but an ant is close by so that the parasitoid, it appears, is unable to reach it. Another ant arrives from near the base of the leaf and scares away the wasp.
(MPG)
