# Supplementary figures and images for: Ecological Complexity in a Coffee Agroecosystem: Spatial Heterogeneity, Population Persistence and Biological Control
Source: PLoS One. 2012 Sep 20;7(9):e45508. doi: 10.1371/journal.pone.0045508 (PMC3447771; doi:10.1371/journal.pone.0045508)

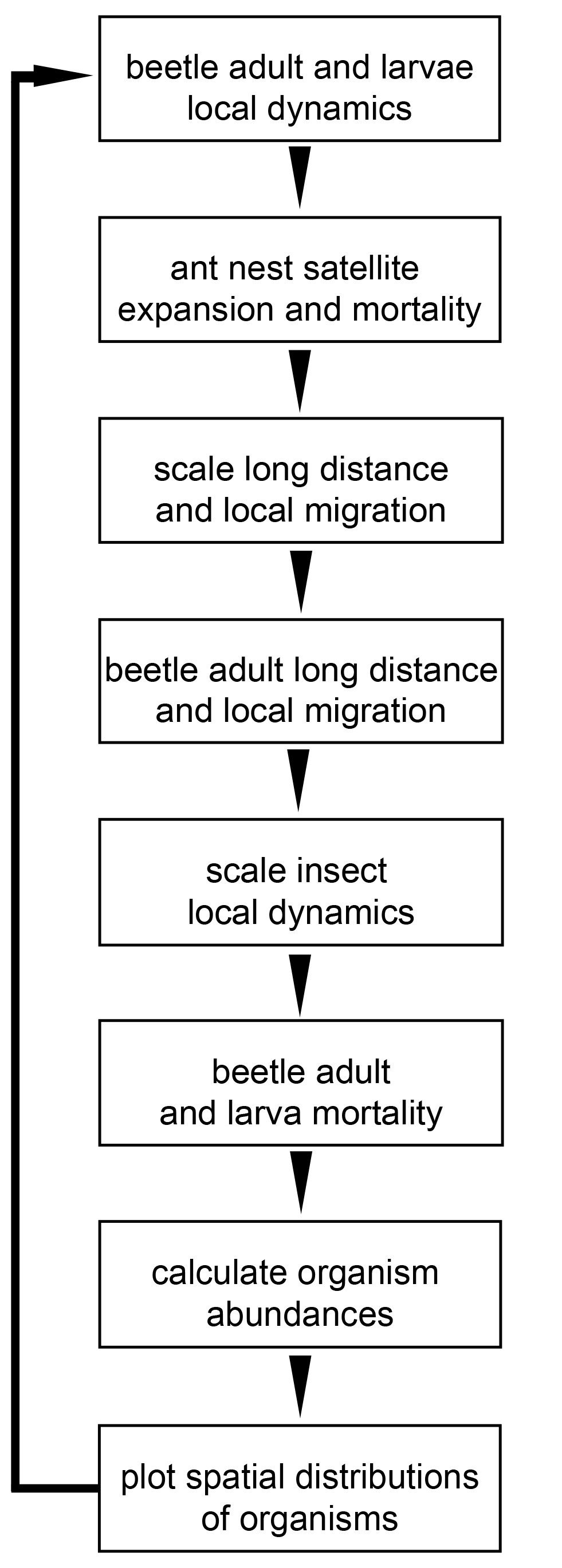

Supplement: Figure S1 — Flow chart of model execution. (TIF) [file pone.0045508.s001.tif]

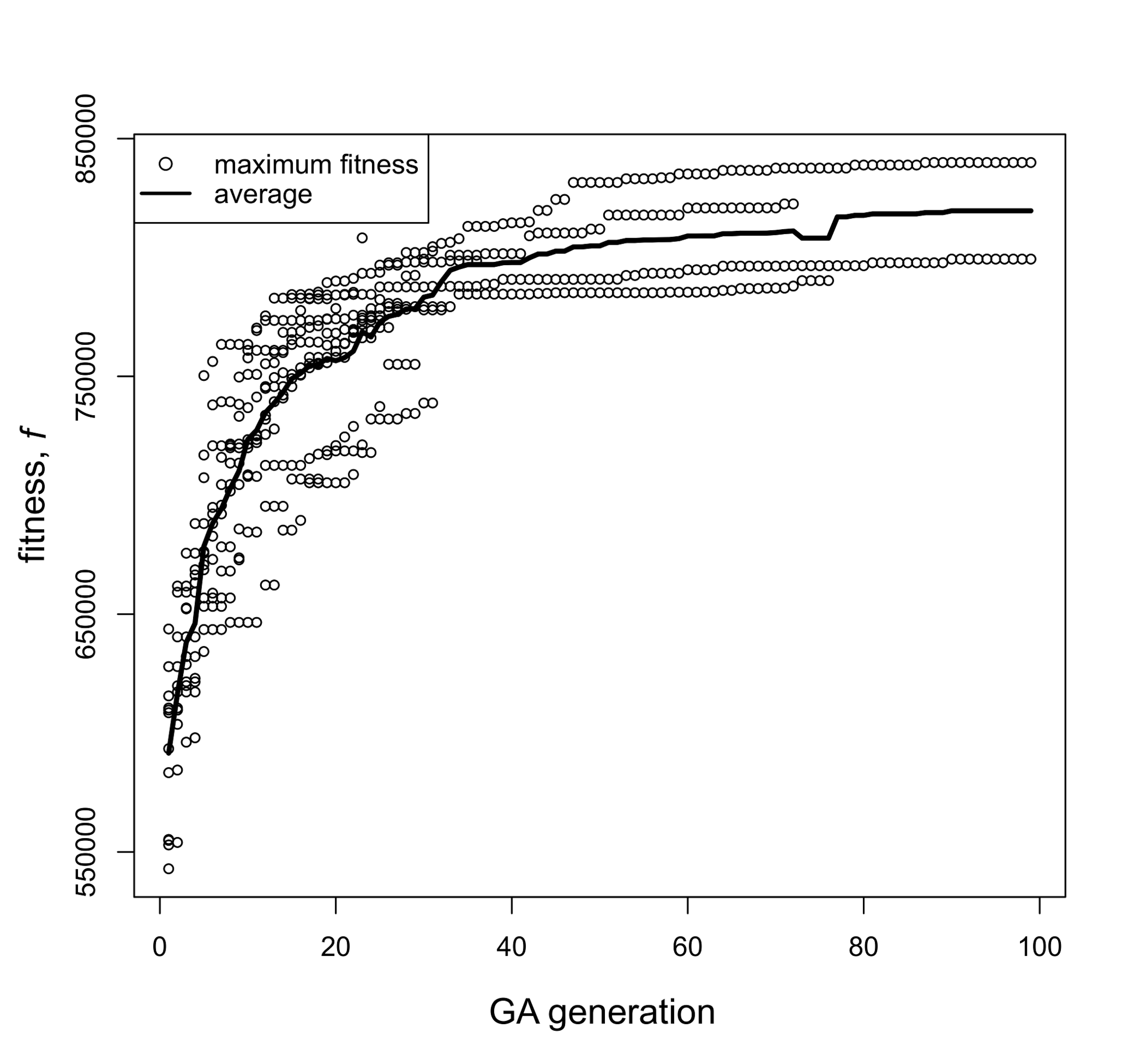

Supplement: Figure S2 — Fitness versus generation for 12 instantiations of the Genetic Algorithm. (TIFF) [file pone.0045508.s002.tiff]
