# Supplementary material for: Ecological Complexity in a Coffee Agroecosystem: Spatial Heterogeneity, Population Persistence and Biological Control
Source: PLoS One. 2012 Sep 20;7(9):e45508. doi: 10.1371/journal.pone.0045508 (PMC3447771; doi:10.1371/journal.pone.0045508)
Supplement: Table S1 — Genetic Algorithm fitness function coefficients. (DOCX) [file pone.0045508.s003.docx]

Table S1. Genetic Algorithm fitness function coefficients

| Coefficient | Description | Value |
| --- | --- | --- |
| *F_min_* | minimum fitness contribution before weighting | 0 |
| *F_max_* | maximum fitness contribution before weighting | 10100000 |
| *n* | total number of performance criteria | 15 |
| *target_x_1_* | number of ant nests (dynamic ants) | 300 |
| *target_x_2_* | number of adult beetles per cell in patches with ants (dynamic ants) | 5.76 |
| *target_x_3_* | number of adult beetles per cell in patches without ants (dynamic ants) | 1.27 |
| *target_x_4_* | number of beetle larvae per cell in patches with ants (dynamic ants) | 3.8 |
| *target_x_5_* | number of beetle larvae per cell in patches without ants (dynamic ants) | 1.58 |
| *target_x_6_* | number of ant nests (all ants) | 300 |
| *target_x_7_* | number of adult beetles per cell in patches with ants (all ants) | 0 |
| *target_x_8_* | number of adult beetles per cell in patches without ants (all ants) | 0 |
| *target_x_9_* | number of beetle larvae per cell in patches with ants (all ants) | 0 |
| *target_x_10_* | number of beetle larvae per cell in patches without ants (all ants) | 0 |
| *target_x_11_* | number of ant nests (no ants) | 300 |
| *target_x_12_* | number of adult beetles per cell in patches with ants (no ants) | 0 |
| *target_x_13_* | number of adult beetles per cell in patches without ants (no ants) | 0 |
| *target_x_14_* | number of beetle larvae per cell in patches with ants (no ants) | 0 |
| *target_x_15_* | number of beetle larvae per cell in patches without ants (no ants) | 0 |
| *min_x_1_* | minimum number of ant nests (dynamic ants) | 1 |
| *min_x_2_* | minimum number of adult beetles per cell in patches with ants (dynamic ants) | 0.001 |
| *min_x_3_* | minimum number of adult beetles per cell in patches without ants (dynamic ants) | 0.001 |
| *min_x_4_* | minimum number of beetle larvae per cell in patches with ants (dynamic ants) | 0.001 |
| *min_x_5_* | minimum number of beetle larvae per cell in patches without ants (dynamic ants) | 0.001 |
| *min_x_6_* | minimum number of ant nests (all ants) | 1 |
| *min_x_7_* | minimum number of adult beetles per cell in patches with ants (all ants) | -1 |
| *min_x_8_* | minimum number of adult beetles per cell in patches without ants (all ants) | -1 |
| *min_x_9_* | minimum number of beetle larvae per cell in patches with ants (all ants) | -1 |
| *min_x_10_* | minimum number of beetle larvae per cell in patches without ants (all ants) | -1 |
| *min_x_11_* | minimum number of ant nests (no ants) | 1 |
| *min_x_12_* | minimum number of adult beetles per cell in patches with ants (no ants) | -1 |
| *min_x_13_* | minimum number of adult beetles per cell in patches without ants (no ants) | -1 |
| *min_x_14_* | minimum number of beetle larvae per cell in patches with ants (no ants) | -1 |
| *min_x_15_* | minimum number of beetle larvae per cell in patches without ants (no ants) | -1 |
| *max_x_1_* | maximum number of ant nests (dynamic ants) | 11000 |
| *max_x_2_* | maximum number of adult beetles per cell in patches with ants (dynamic ants) | 10000000 |
| *max_x_3_* | maximum number of adult beetles per cell in patches without ants (dynamic ants) | 10000000 |
| *max_x_4_* | maximum number of beetle larvae per cell in patches with ants (dynamic ants) | 10000000 |
| *max_x_5_* | maximum number of beetle larvae per cell in patches without ants (dynamic ants) | 10000000 |
| *max_x_6_* | maximum number of ant nests (all ants) | 11000 |
| *max_x_7_* | maximum number of adult beetles per cell in patches with ants (all ants) | 2 |
| *max_x_8_* | maximum number of adult beetles per cell in patches without ants (all ants) | 2 |
| *max_x_9_* | maximum number of beetle larvae per cell in patches with ants (all ants) | 2 |
| *max_x_10_* | maximum number of beetle larvae per cell in patches without ants (all ants) | 2 |
| *max_x_11_* | maximum number of ant nests (no ants) | 11000 |
| *max_x_12_* | maximum number of adult beetles per cell in patches with ants (no ants) | 2 |
| *max_x_13_* | maximum number of adult beetles per cell in patches without ants (no ants) | 2 |
| *max_x_14_* | maximum number of beetle larvae per cell in patches with ants (no ants) | 2 |
| *max_x_15_* | maximum number of beetle larvae per cell in patches without ants (no ants) | 2 |
| *w_1_* | weight for number of ant nests (dynamic ants) | 0.11 |
| *w_2_* | weight for number of adult beetles per cell in patches with ants (dynamic ants) | 0.11 |
| *w_3_* | weight for number of adult beetles per cell in patches without ants (dynamic ants) | 0.11 |
| *w_4_* | weight for number of beetle larvae per cell in patches with ants (dynamic ants) | 0.11 |
| *w_5_* | weight for number of beetle larvae per cell in patches without ants (dynamic ants) | 0.11 |
| *w_6_* | weight for number of ant nests (all ants) | 0 |
| *w_7_* | weight for number of adult beetles per cell in patches with ants (all ants) | 0.11 |
| *w_8_* | weight for number of adult beetles per cell in patches without ants (all ants) | 0 |
| *w_9_* | weight for number of beetle larvae per cell in patches with ants (all ants) | 0.11 |
| *w_10_* | weight for number of beetle larvae per cell in patches without ants (all ants) | 0 |
| *w_11_* | weight for number of ant nests (no ants) | 0 |
| *w_12_* | weight for number of adult beetles per cell in patches with ants (no ants) | 0 |
| *w_13_* | weight for number of adult beetles per cell in patches without ants (no ants) | 0.11 |
| *w_14_* | weight for number of beetle larvae per cell in patches with ants (no ants) | 0 |
| *w_15_* | weight for number of beetle larvae per cell in patches without ants (no ants) | 0.11 |
