# Supplementary material for: Ecological Complexity in a Coffee Agroecosystem: Spatial Heterogeneity, Population Persistence and Biological Control
Source: PLoS One. 2012 Sep 20;7(9):e45508. doi: 10.1371/journal.pone.0045508 (PMC3447771; doi:10.1371/journal.pone.0045508)
Supplement: Table S2 — Parameter values used for the model. (DOCX) [file pone.0045508.s004.docx]

Table S2. Parameter values used for the model

|  | | |
| --- | --- | --- |
| Parameter | Description | Value |
| *x_0_* | intercept of ant nest density-dependent local expansion function | 2.0 X 10^-7^ |
| *x_1_* | slope of ant nest density-dependent local expansion function | 0.7365 |
| *d_0_* | intercept of ant nest scale-dependent mortality function | 0.82 |
| *d_1_* | slope of ant nest scale-dependent mortality function | 0.3 |
| *v_0_* | intercept of scale density-dependent local migration function | 0 |
| *v_1_* | slope of scale density-dependent local migration function | 0.7129 |
| *w* | probability of a cell receiving propagule rain of scales | 1.0 X 10^-3^ |
| *p_X_* | coefficient of propagule rain of scales | 2.0 X 10^-4^ |
| *m_1_* | coefficient of local migration of scales | 0.02 |
| *r_(A)_* | intrinsic growth rate of scales with ants | 1.9968 |
| *r_(noA)_* | intrinsic growth rate of scales without ants | 1.5938 |
| *c_L_* | predation rate of larval beetles with or without ants | 0.7568 |
| *c_A(A)_* | predation rate of adult beetles with ants | 0.0107 |
| *c_A(noA)_* | predation rate of adult beetles without ants | 0.3231 |
| *g* | functional response of larval and adult beetles | 2.0989 |
| *e* | eclosion rate of beetles | 1 |
| *b* | birth rate of beetles | 962.7603 |
| *s_A(A)_* | survivorship rate of adult beetles with ants | 0.4690 |
| *s_A(noA)_* | survivorship rate of adult beetles without ants | 0.8841 |
| *s_L(A)_* | survivorship rate of larval beetles with ants | 0.9345 |
| *s_L(noA)_* | survivorship rate of larval beetles without ants | 0.6341 |
| *a_o_* | intercept of adult beetle density-dependent local migration function | 0 |
| *a_1_* | slope of adult beetle density-dependent local migration function | 0.5 |
| *m_2_* | coefficient of local migration of adult beetles | 0.8914 |
| *p_A_* | coefficient of propagule rain of adult beetles | 0.1129 |
| *u_(A)_* | probability of a cell with ants receiving propagule rain of beetle adults | 0.2332 |
| *u_(noA)_* | probability of a cell without ants receiving propagule rain of beetle adults | 0.1994 |
