# Supplementary material for: Ecological Complexity in a Coffee Agroecosystem: Spatial Heterogeneity, Population Persistence and Biological Control
Source: PLoS One. 2012 Sep 20;7(9):e45508. doi: 10.1371/journal.pone.0045508 (PMC3447771; doi:10.1371/journal.pone.0045508)
Supplement: Methods S1 — Model pseudo-code. (DOCX) [file pone.0045508.s005.docx]

## Methods S1. Model pseudo-code

## Initialization

For comparison purposes, we followed Vandermeer et al [12] initiation process; our model, however, is robust to changes in initiation details. Following the initiation process of their model, each cell in the initial ant nest matrix is populated randomly with probability 0.16. The scale insect matrix is populated by drawing a number, *randn*, for each cell from a normal distribution with mean 0 and standard deviation one; if *randn <* -1, the cell is populated with |*randn*| scales, otherwise the cell remains unpopulated. The beetle adult and larvae matrices are populated similarly, but for biological accuracy (since beetle numbers are much lower than scale numbers), the *randn* threshold is -2 for both.

Thus the model starts with randomly scattering ant nests (ones and zeros), scales and beetles all over the lattice. Given these initial conditions, the average initial number of organisms used was:

Ant nests: 241 (of a total of 10800 cells)

Scales: 1680 cells populated with an average of 0.16 scales per cell

Beetle adults: 241 cells populated with an average of 0.0228 adults per cell

Beetle larvae: 241 cells populated with an average of 0.0228 larvae per cell

The model then followed the code below and with the sequence outlined in Fig. SM 1

## Submodels

Beetle adult local dynamics:

FOR all cells in beetle adult grid

IF with ants THEN

*A_t+1_*’ = (*e***c_L(A_*_)_**L_t_***N_t_*)/(1+*g***N_t_*)

ELSE

*A_t+1_*’ = (*e***c_L(noA)_***L_t_***N_t_*)/(1+g**N_t_*)

END IF

END FOR

Beetle larvae local dynamics:

FOR all cells in beetle larvae grid

IF with ants THEN

*L_t+1_*’ = (*b***c_A(A)_***A_t_***N_t_*)/(1+*g***N_t_*)

ELSE

*L_t+1_*’ = (*b***c_A(noA)_***A_t_***N_t_*)/(1+*g***N_t_*)

END IF

END FOR

Ant nest satellite expansion

*D_t, Moore_* = number of ant nests in the Moore neighborhood at time *t*

FOR all cells in ant nest grid

*rand* = random number between 0 and 1 drawn from a uniform distribution

IF cell is not occupied by an ant nest THEN

IF *rand < x_o_ + x_1_*D_t, Moore_*

cell becomes occupied by an ant nest

END IF

END IF

END FOR

Ant nest mortality

FOR all cells in ant nest grid

*rand* = random number between 0 and 1 drawn from a uniform distribution

IF cell is occupied by an ant nest THEN

IF *rand < d_o_ - d_1_*N_t_*

cell becomes unoccupied

END IF

END IF

END FOR

Scale long distance migration:

*propagule_rain_count* = 0

FOR all cells in scale grid

*N_t+_*_1_’ = *N_t_*

*rand* = random number between 0 and 1 drawn from a uniform distribution

IF *rand < w* THEN

mark cell to receive scale propagule rain

*propagule_rain_count* = *propagule_rain_count +* 1

END IF

END FOR

*propagule_rain* = *p_x_**(total # of scales in grid)/*propagule_rain_count*

FOR all cells marked to receive propagule rain

*N_t+_*_1_’ = *N_t_ + propagule_rain*

END FOR

Scale local migration:

*N_t, Moore_* = number of scales in the Moore neighborhood at time *t*

FOR all cells in scale grid

*rand* = random number between 0 and 1 drawn from a uniform distribution

IF *rand < v_0_ + v_1_*N_t, Moore_* THEN

*local_migration* = 0

FOR all cells in Moore neighborhood of cell

*N_t+_*_1_’ = *N_t+_*_1_’ - *m_1_*N_t_*

*local_migration* = *local_migration + m_1_ *N_t_*

END FOR

*N_t+_*_1_’ = *N_t+_*_1_’ + *local_migration*

END IF

END FOR

FOR all cells in scale grid

*N_t+_*_1_’ = *N_t+_*_1_’*(1-*p_x_*)

END FOR

Beetle adult long distance migration:

*propagule_rain_count* = 0

FOR all cells in beetle adult grid

*A_t+_*_1_’’ = *A_t+1_’*

*rand* = random number between 0 and 1 drawn from a uniform distribution

IF with ants THEN

IF *rand < u_(A)_* THEN

mark cell to receive beetle adult propagule rain

*propagule_rain_count* = *propagule_rain_count +* 1

END IF

ELSE

IF *rand < u_(noA)_* THEN

mark cell to receive beetle adult propagule rain

*propagule_rain_count* = *propagule_rain_count +* 1

END IF

END FOR

*propagule_rain* = *p_A_**(total # of beetle adults in grid)/*propagule_rain_count*

FOR all cells marked to receive propagule rain

*A_t+_*_1_’’ = *A_t+1_’ + propagule_rain*

END FOR

Beetle adult local migration:

*A_t+1_’_, Moore_* = number of beetle adults in the Moore neighborhood, using *A_t+1_’*

FOR all cells in beetle adult grid

*rand* = random number between 0 and 1 drawn from a uniform distribution

IF *rand < a_0_ + a_1_*A_t+1_’_, Moore_* THEN

mark cell as a sink

END IF

END FOR

FOR all cells in beetle adult grid

IF cell marked as a sink THEN

*local_migration* = 0

FOR all cells in Moore neighborhood of cell

*migration_fraction* =

min(*m_2_,* 1/number of sinks in source cell’s Moore neighborhood)

*A_t+_*_1_’’ = *A_t+_*_1_’’ - *migration_fraction*A_t+_*_1_’

*local_migration* = *local_migration + migration_fraction*A_t+_*_1_’

END FOR

*A_t+_*_1_’’ = *A_t+_*_1_’’ + *local_migration*

END IF

END FOR

FOR all cells in beetle adult grid

*A_t+_*_1_’’ = *A_t+_*_1_’’*(1-*p_A_*)

END FOR

Scale insect local dynamics:

FOR all cells in scale grid

IF with ants THEN

*N_t+1_* =

*r_(A)_*N_t+1_’**(1-*N_t+1_’*)

- (*c_A(A)_*)*(*A_t+_*_1_’’)*(*N_t+1_’*)/(1+*g***N_t+1_’*)

- (*c_L_*)*(*L_t+1_*’)*(*N_t+1_’*)/(1+*g***N_t+1_’*)

ELSE

*N_t+1_* =

*r_(noA)_*N_t+1_’**(1-*N_t+1_’*)

- (*c_A(noA)_*)*(*A_t+_*_1_’’)*(*N_t+1_’*)/(1+*g***N_t+1_’*)

- (*c_L_*)*(*L_t+1_*’)*(*N_t+1_’*)/(1+*g***N_t+1_’*)

END IF

END FOR

Beetle adult mortality:

FOR all cells in beetle adult grid

IF with ants THEN

*A_t+_*_1_ = *A_t+_*_1_’’**s_A(A)_*

ELSE

*A_t+_*_1_ = *A_t+_*_1_’’**s_A(noA)_*

END IF

END FOR

Beetle larvae mortality:

FOR all cells in beetle larvae grid

IF with ants THEN

*L_t+_*_1_ = *L_t+_*_1_’**s_L(A)_*

ELSE

*L_t+_*_1_ = *L_t+_*_1_’**s_L(noA)_*

END IF

END FOR
