# Supplementary material for: Ecological Complexity in a Coffee Agroecosystem: Spatial Heterogeneity, Population Persistence and Biological Control
Source: PLoS One. 2012 Sep 20;7(9):e45508. doi: 10.1371/journal.pone.0045508 (PMC3447771; doi:10.1371/journal.pone.0045508)
Supplement: Methods S2 — Genetic algorithm (GA) description and parameters. (DOCX) [file pone.0045508.s006.docx]

**Methods S2. Genetic algorithm (GA) description and parameters**

We employed a genetic algorithm (GA) implemented using the Java package JGAP (Java Genetic Algorithms Package) to search for parameter values fulfilling certain performance criteria. The performance (fitness) of parameter value combinations was calculated based on the following criteria: number of ant nests, complete extinction of beetles when ants are fixed to occupy 100% of the lattice points, complete extinction of beetles when ants are fixed to occupy 0% of the lattice points, numbers of beetle adults and larvae in locations with ant nests, and numbers of beetle adults and larvae in locations without ant nests, as follows,

$$f=\sum_{i=1}^{n} \left( w_{i}\left( \left\| \left\{ \begin{matrix} \begin{matrix} F_{min} :x_{i}<min\_x_{i} \\ F_{min}+\left( \frac{x_{i}-min\_x_{i}}{target\_x_{i}-min\_xi} \right)\left( F_{max}-F_{min} \right) :min\_x_{i}<x_{i}<target\_x_{i} \\ F_{max}-\left( \frac{x_{i}-target\_x_{i}}{max\_x_{i}-target\_x_{i}} \right)\left( F_{max}-F_{min} \right) :target\_x_{i}<x_{i}<max\_x_{i} \end{matrix} \\ F_{max} :x_{i}<max\_x_{i} \end{matrix} \right. \right\| \right) \right)$$

where *f* is the fitness; *n* is the total number of performance criteria; and *x_i_* is the value of criterion *i* at the end of the simulation run. For each set of parameters, the model was run three times: once with the ant nests appearing and dying dynamically, as defined by the rules of the cellular automata and coupled map lattice models (dynamic ants), a second time with ants occupying every cell in the lattice (all ants), and a third time with no ant nests (no ants). The values and descriptions of the coefficients are found in Table S1.

In order to find acceptable parameter values more rapidly, the GA was run on 12 computers simultaneously. Each instantiation of the GA had a population of 100 possible solutions, with the parameter values in the first generation chosen at random. The maximum fitness per generation for each of the 12 instantiations is shown in Fig. S2. There was significant variability in the speed of the computers used, so there was substantial variation in the number of generations the machines completed before the search was terminated. All of the instantiations converged rapidly to marginally acceptable or acceptable performance, suggesting that the range of acceptable parameter values is relatively large, i.e., the model output is relatively robust to changes in parameter values.
